# Comparison of Surgical Outcomes Between the Hinotori and da Vinci Platforms in Robot‐Assisted Radical Prostatectomy: A Propensity Score‐Matched Study

**DOI:** 10.1111/ases.70326

**Published:** 2026-06-15

**Authors:** Masanari Nishida, Akinori Wada, Tetsuya Yoshida, Kubota Shigehisa, Masayuki Nagasawa, Kenichi Kobayashi, Kazuaki Yamanaka, Kazuyoshi Johnin, Susumu Kageyama

**Affiliations:** ^1^ Department of Urology Shiga University of Medical Science Otsu Japan

**Keywords:** da Vinci surgical system, hinotori surgical robot system, positive surgical margin, prostate cancer, robot‐assisted radical prostatectomy, urinary continence recovery

## Abstract

**Introduction:**

Comparative evidence between robotic platforms for robot‐assisted radical prostatectomy (RARP) remains limited, particularly regarding oncological quality beyond perioperative outcomes. This study compared outcomes between the hinotori and da Vinci systems, focusing on positive surgical margin (PSM) distribution in a real‐world cohort with varying surgeon experience.

**Methods:**

We reviewed 437 patients who underwent RARP at our institution between 2013 and 2025. After propensity score matching, 130 patients in the da Vinci group and 65 in the hinotori group were analyzed. Perioperative outcomes, PSM rates and anatomical distribution, urinary continence recovery, and biochemical recurrence (BCR)‐free survival were compared.

**Results:**

The matched cohort involved 17 surgeons, including 12 hinotori surgeons, with expert surgeons performing 38% of hinotori procedures. Operative, console, and setup‐related times were longer, and estimated blood loss was greater in the hinotori group. No transfusions were required. Complication rates and hospital stay were comparable. No differences were observed in overall or stage‐specific PSM rates. PSMs most frequently involved the apex and posterolateral regions in both groups, with no difference in anatomical PSM distribution. The 12‐month urinary continence recovery rates were 87% in the hinotori group and 85% in the da Vinci group. In the 2‐year time‐aligned analysis, BCR‐free survival rates in the hinotori and da Vinci groups were 94% and 89% at 1 year and 89% and 85% at 2 years, respectively.

**Conclusion:**

Hinotori‐assisted RARP showed longer operative and setup‐related times but no apparent deterioration in perioperative safety, PSM outcomes including anatomical distribution, continence recovery, or early BCR‐free survival.

## 1 Introduction

Prostate cancer (PCa) is among the most commonly diagnosed malignancies in men. According to GLOBOCAN 2022, approximately 1.4 million new cases and 396 000 PCa‐related deaths were reported globally [1]. Radical prostatectomy forms the cornerstone of curative treatment for patients with localized or locally advanced PCa. Over the past two decades, robot‐assisted radical prostatectomy (RARP) has become the most widely adopted surgical approach and is considered the standard of care for localized PCa, offering improved visualization and instrumentation dexterity compared with open or conventional laparoscopy [2]. The da Vinci surgical system (Intuitive Surgical, Sunnyvale, CA, USA) has been instrumental in cementing the central role of robotic surgery in urology. In recent years, several new robotic surgical platforms have been developed and introduced into clinical practice. These include the hinotori (Medicaroid Corporation, Kobe, Japan), Revo‐i (Meerecompany Inc., Seongnam, Republic of Korea), Senhance (Asensus Surgical, Durham, NC, USA), Versius (CMR Surgical, Cambridge, UK), and Hugo RAS surgical systems (Medtronic, Minneapolis, MN, USA) [3, 4]. Consequently, RARP can now be performed using multiple robotic platforms worldwide, raising important questions regarding the potential differences in surgical and oncological outcomes among these systems.

Hinotori, the first domestically developed surgical robotic system in Japan, differs from the da Vinci platform in several structural aspects, including an additional degree of freedom provided by eight‐axis robotic arms, software‐based trocar positioning without physical docking, and a fully adjustable high‐definition 3D viewer, which may improve flexibility and reduce surgeon fatigue [5, 6]. Early clinical reports have described the feasibility and perioperative characteristics of hinotori‐assisted RARP, particularly longer operative and console times during the initial adoption phase [7, 8, 9]. However, comparative evidence regarding oncological quality remains limited, particularly with respect to positive surgical margin (PSM) rates, anatomical PSM distribution, and BCR‐free survival. In addition, the influence of surgeon heterogeneity during the introduction of a new robotic platform remains an important consideration in real‐world clinical practice. Therefore, the present study aimed to compare perioperative, pathological, functional, and early oncological outcomes between the hinotori and da Vinci systems using a propensity score‐matched real‐world cohort involving 17 surgeons with varying levels of RARP experience. Particular emphasis was placed on PSM rates and anatomical distribution, urinary continence recovery, and early BCR‐free survival.

## 2 Patients and Methods

### 2.1 Study Design and Patients

This single‐center, retrospective cohort study was conducted at Shiga University of Medical Science Hospital. We reviewed the medical records of consecutive patients who underwent RARP for PCa between May 2013 and March 2025. The study protocol was approved by the Institutional Review Board of Shiga University of Medical Science Hospital (No. R2017‐034). During the study period, RARP was performed using two robotic surgical platforms: the da Vinci Si or Xi surgical system and hinotori surgical robot system. The da Vinci system was used throughout the study period, whereas the hinotori system was introduced at our institution in May 2022 and used thereafter. A total of 439 patients underwent RARP, of which 367 patients were treated with the da Vinci system and 72 patients with the hinotori system. Patients were eligible for inclusion if they had histologically confirmed PCa and underwent RARP as primary treatment. In the hinotori group, one patient with a pathological diagnosis of sarcoma and one patient who required intraoperative conversion to the da Vinci system because of mechanical malfunction were excluded. Consequently, the final study cohort comprised 437 patients: 367 in the da Vinci group and 70 in the hinotori group. The choice of the robotic platform was determined primarily by the period of surgery and institutional availability, rather than patient characteristics.

### 2.2 Surgical Procedure

In the propensity score‐matched cohort, RARP procedures were performed by 17 surgeons overall, including 12 surgeons who performed hinotori‐assisted procedures. All surgeons performing hinotori‐assisted procedures were certified to operate the hinotori robotic system and had prior experience in RARP. Expert status was defined based on the number of prior RARP cases performed before the introduction of the hinotori system, with ≥ 40 prior cases classified as expert surgeons. The transperitoneal anterior approach was used for all procedures in both groups. Standardized surgical steps, including bladder neck dissection, seminal vesicle mobilization, control of the prostatic pedicles, apical dissection, and urethrovesical anastomosis, were used for both platforms, as reported previously [10]. Nerve‐sparing was performed at the discretion of the surgeon, based on preoperative risk stratification, tumor characteristics, and patient preference, and was categorized as none, unilateral, or bilateral. Pelvic lymph node dissection was performed based on preoperative risk assessment, in accordance with contemporary guideline recommendations and institutional policies at the time of surgery.

### 2.3 Data Collection

The baseline patient characteristics included age, body mass index (BMI), serum prostate‐specific antigen (PSA) level at diagnosis, prostate volume measured using transrectal ultrasonography or magnetic resonance imaging (MRI), biopsy Gleason grade group, clinical T stage, National Comprehensive Cancer Network (NCCN) risk group, use of neoadjuvant hormonal therapy, and history of abdominal surgery. Perioperative variables comprised operative and console times; port placement time; docking time; time to console start; estimated blood loss (EBL); blood transfusion (considered based on intraoperative blood loss, postoperative hemoglobin levels, and hemodynamic status); nerve‐sparing procedure (none, unilateral, or bilateral); pelvic lymph node dissection (performed or not); intraoperative complications; major postoperative complications, defined as Clavien–Dindo grade ≥ 3 occurring within 30 days after surgery, as adjudicated by the attending surgeons; and length of postoperative hospital stay. Port placement time was defined as the interval from skin incision to robot roll‐in. Docking time was defined as the interval from robot roll‐in to the start of console operation, thereby including docking and preconsole preparation. Time to console start was defined as the interval from skin incision to the start of console operation. The pathological outcomes included the pathological Gleason grade, pathological T stage, and surgical margin status. A PSM was defined as the presence of tumor cells on the inked surface of the surgical specimen. In a previous study [11], PSM locations were categorized into six anatomical sites: the apex, bladder neck, anterior, posterolateral, posterior, and seminal vesicles. Cases with PSMs at two or more locations were classified as multifocal. The postoperative functional outcomes focused on the recovery of urinary continence, defined as the use of 0 or 1 safety pad per day. Continence status was assessed based on patient‐reported pad use. Assessments were performed daily during the initial postoperative period until discharge, at 1 month after surgery, and every 3 months thereafter. The time to recovery of urinary continence was calculated from the date of surgery to the first visit at which continence was achieved. Patients who did not recover during follow‐up were censored at the last follow‐up visit. No missing data were observed for continence outcomes. The date of BCR was defined as the date of the first PSA value ≥ 0.2 ng/mL, which was subsequently confirmed by a second consecutive measurement. Serum PSA levels were measured every 3–6 months during follow‐up according to the institutional protocol. The time to BCR was calculated from the date of surgery to the date of BCR. Patients whose serum PSA levels did not decrease to < 0.2 ng/mL after surgery were considered to have BCR at time zero. Patients without BCR were censored at the date of last follow‐up. Patients who received salvage therapy before documented BCR were censored at the time of initiation of salvage therapy. The postoperative follow‐up duration was recorded in months.

### 2.4 Propensity Score Matching

To mitigate selection bias and confounding due to the nonrandomized nature of treatment allocation, propensity score matching was performed using a multivariable logistic regression model. The covariates included in the propensity score model were age, BMI, serum PSA level at diagnosis, prostate volume, clinical T stage, biopsy Gleason grade group, use of neoadjuvant hormonal therapy, history of abdominal surgery, nerve‐sparing, and pelvic lymph node dissection. Patients were matched at a ratio of 2:1 (da Vinci:hinotori) using a nearest‐neighbor matching algorithm without replacement. A caliper width of 0.2 of the standard deviation of the logit of the propensity score was applied. The balance between the matched groups was assessed using both *p* values and standardized mean differences (SMDs) for each covariate. An absolute SMD of < 0.1 was designated adequate covariate balance.

### 2.5 Statistical Analysis

Continuous variables were presented as medians with interquartile ranges (IQRs) and compared using the Mann–Whitney U test. Categorical variables were expressed as numbers and percentages and compared using the chi‐squared test or Fisher's exact test, as appropriate. The time to urinary continence recovery and BCR‐free survival were analyzed using the Kaplan–Meier method, and differences between the groups were assessed using the log‐rank test. To account for differences in follow‐up duration between groups, a time‐aligned analysis with administrative censoring at 2 years (24 months) was performed for BCR‐free survival. Univariable and multivariable logistic regression analyses were performed to identify the predictors of PSM in the propensity score‐matched cohort. Surgery year was included as a continuous variable in the regression models to account for potential era effects. All statistical analyses were performed using EZR version 1.70 (Saitama Medical Center, Jichi Medical University, Saitama, Japan), a graphical user interface for R version 4.5.2 (The R Foundation for Statistical Computing, Vienna, Austria). Statistical significance was defined as a two‐sided *p* value of < 0.05.

## 3 Results

### 3.1 Patient Characteristics

**FIGURE 1:**
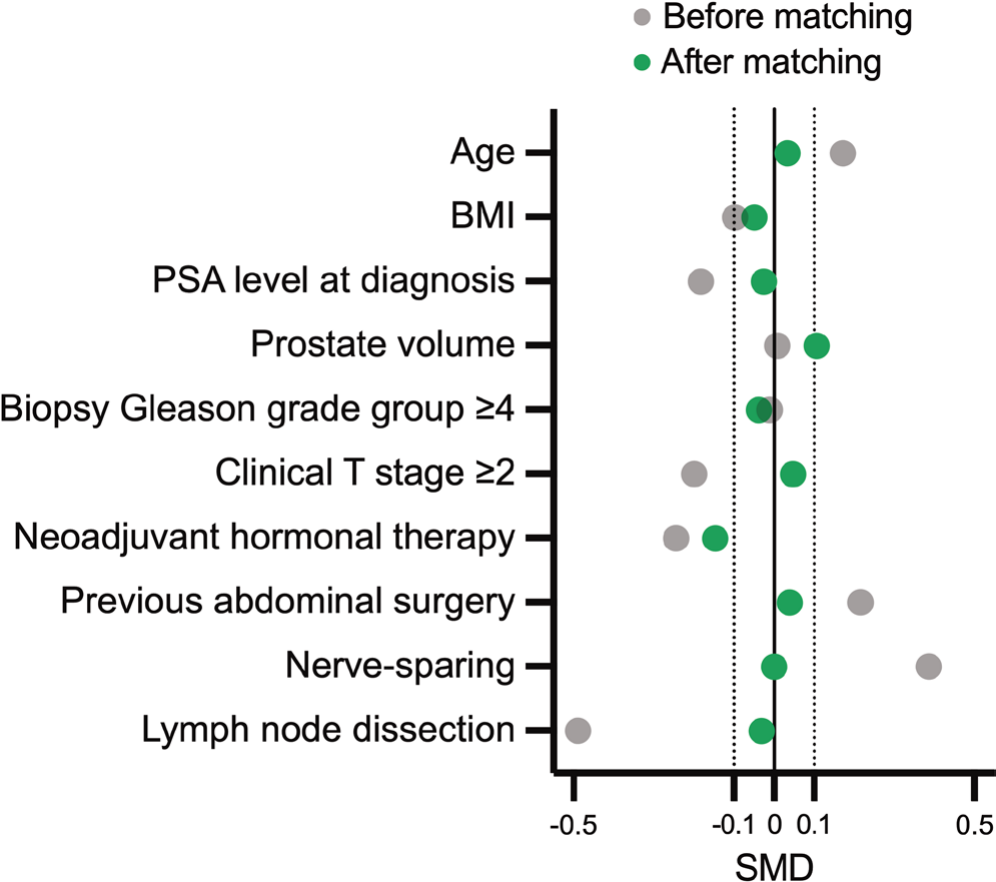
Covariate balance between the da Vinci and hinotori groups before and after propensity score matching. The gray dots indicate standardized mean differences (SMDs) before propensity score matching and the green dots indicate those after matching. Vertical dotted lines represent SMD of ±0.1, indicating adequate balance between the groups.

Baseline patient characteristics before and after propensity score matching are summarized in Table 1. In the entire cohort, patients in the hinotori group tended to be older than those in the da Vinci group. Other baseline variables were generally comparable between the groups, although significant differences were observed in surgical factors, including nerve‐sparing and pelvic lymph node dissection. After propensity score matching, 130 and 65 patients were included in the da Vinci and hinotori groups, respectively. All baseline and surgical variables were well‐balanced between the groups, with no statistically significant differences. The proportions of nerve‐sparing (82% in both groups) and pelvic lymph node dissection (52% in the hinotori group vs. 51% in the da Vinci group) were comparable. Covariate balance was further confirmed by SMDs, which demonstrated overall satisfactory balance across variables (Figure 1).

**TABLE 1:** Baseline comparison of patient characteristics before and after propensity score matching.

	Entire cohort				Propensity score‐matched cohort			
	da Vinci (*N* = 367)	hinotori (*N* = 70)	*p*	SMD	da Vinci (*n* = 130)	hinotori (*n* = 65)	*p*	SMD
Age (years)	68 (64–71)	70 (65–73)	0.060	0.173	69 (65–72)	70 (64–73)	0.436	0.034
BMI (kg/m^2^)	23.5 (22.0–25.4)	23.2 (22.1–25.4)	0.628	−0.097	23.3 (21.8–25.0)	23.3 (22.2–25.4)	0.461	−0.049
PSA (ng/mL)	7.6 (5.5–11.1)	7.1 (4.9–10.4)	0.242	−0.182	7.2 (5.2–10.3)	7.2 (5.0–10.5)	0.986	−0.026
Prostate volume (mL)	28 (21–38)	28 (22–38)	0.942	0.009	26 (21–36)	28 (22–38)	0.349	0.106
Biopsy Gleason grade group, *n* (%)			0.472	−0.011			0.258	−0.038
Grade group 1	49 (13)	8 (11)			23 (15)	7 (11)		
Grade group 2	138 (38)	33 (47)			43 (33)	30 (46)		
Grade group 3	103 (28)	15 (21)			36 (25)	15 (23)		
Grade group 4	45 (12)	6 (9)			17 (13)	5 (8)		
Grade group 5	32 (9)	8 (11)			11 (8)	8 (12)		
Clinical T stage, *n* (%)			0.415	−0.199			0.927	0.048
cT1	112 (31)	28 (40)			51 (39)	24 (37)		
cT2	244 (66)	41 (59)			76 (58)	40 (62)		
cT3	10 (3)	1 (1)			2 (2)	1 (2)		
cT4	1 (< 1)	0 (0)			1 (1)	0 (0)		
NCCN risk group, *n* (%)			0.798	N/A			0.354	N/A
Low	34 (9)	6 (9)			19 (15)	5 (8)		
Intermediate	239 (65)	50 (71)			79 (61)	47 (72)		
High	88 (24)	13 (19)			30 (23)	12 (18)		
Very high	6 (2)	1 (1)			2 (2)	1 (1)		
Neoadjuvant hormonal therapy, *n* (%)	31 (8)	2 (3)	0.138	−0.244	8 (6)	2 (3)	0.501	−0.147
Previous abdominal surgery, *n* (%)	62 (17)	18 (26)	0.092	0.217	24 (18)	13 (20)	0.847	0.039
Nerve‐sparing, *n* (%)			0.002	0.387			0.834	0
None	141 (38)	12 (17)			24 (18)	12 (18)		
Unilateral	165 (45)	41 (59)			73 (56)	39 (60)		
Bilateral	61 (17)	17 (24)			33 (25)	14 (22)		
Lymph node dissection, *n* (%)	252 (69)	35 (50)	0.004	−0.489	66 (51)	34 (52)	0.880	−0.031

*Note:* Values are presented as median (interquartile range [IQR]) or number (%). Biopsy Gleason grade group, clinical T stage, and nerve‐sparing procedure were dichotomized (≥ cT2 vs. cT1, ≥ grade group 4 vs. ≤ 3, and yes vs. no, respectively) for the calculation of standardized mean differences (SMDs).

Abbreviations: BMI, body mass index; NCCN, National Comprehensive Cancer Network; PSA, prostate‐specific antigen.

The distribution of cases across surgeons is shown in Table 2. While several surgeons were classified as experts in RARP at the time of hinotori introduction, their involvement in hinotori‐assisted procedures was relatively limited, with only 25 of 65 cases (38%) performed by expert surgeons.

**TABLE 2:** Distribution of cases by surgeon and robotic platform in the propensity score‐matched cohort, stratified by surgeon experience at the time of hinotori introduction.

Surgeon	Total (*n* = 195)	da Vinci (*n* = 130)	hinotori (*n* = 65)	Experience^a^
A	32	30	2	Expert
B	25	15	10	Expert
C	23	19	4	Expert
D	16	16	0	Nonexpert
E	15	9	6	Expert
F	14	11	3	Expert
G	10	1	9	Nonexpert
H	9	1	8	Nonexpert
I	8	8	0	Expert
J	8	8	0	Nonexpert
K	8	0	8	Nonexpert
L	7	7	0	Expert
M	7	0	7	Nonexpert
N	5	5	0	Nonexpert
O	5	0	5	Nonexpert
P	2	0	2	Nonexpert
Q	1	0	1	Nonexpert

*Note:* Values are presented as number of cases.

^a^ Surgeons with ≥ 40 prior RARP cases at the time of hinotori introduction were defined as expert surgeons.

### 3.2 Perioperative and Pathological Outcomes

The perioperative and pathological outcomes in the propensity score‐matched cohort are shown in Table 3. The operative and console times were significantly longer in the hinotori group than in the da Vinci group (311 vs. 252 min, *p* < 0.001; 232 vs. 188 min, *p* < 0.001, respectively). In addition, setup‐related times were also significantly longer in the hinotori group, including port placement time (25 vs. 21 min, *p* < 0.001), docking time (11 vs. 7 min, *p* < 0.001), and time to console start (37 vs. 29 min, *p* < 0.001). The EBL was also significantly greater in the hinotori group (200 vs. 100 mL, *p* < 0.001). None of the patients in either group required blood transfusion. The rates of intraoperative and major postoperative complications (Clavien–Dindo grade ≥ 3) were low in both groups. The length of postoperative hospital stay was comparable between the groups (median, 9 days for both; *p* = 0.842). Regarding the pathological outcomes, no significant differences were observed in the pathological Gleason grade group distribution or pathological T stage (*p* = 0.647 and *p* = 0.240, respectively) between the groups. The overall PSM rate was numerically higher in the hinotori group than in the da Vinci group (35% vs. 28%), although the difference lacked statistical significance (*p* = 0.323). When stratified by pathological stage, the rates of PSM among patients with pT2 disease were 30% (13/44) in the hinotori group and 19% (17/91) in the da Vinci group (*p* = 0.182). Among patients with pT3 or pT4 disease, the PSM rates were similarly high in both groups (48% [10/21] vs. 51% [20/39], *p* = 1.000). The rate of lymph node involvement was comparable between the groups (12% [4/34] vs. 8% [5/66], *p* = 0.485). The distribution of PSM locations in the propensity score‐matched cohort is shown in Table 4. The most frequent locations of PSMs were the apex and posterolateral regions in both groups. The proportions of apical PSMs were similar between the groups (26% vs. 32%), as were those of posterolateral PSMs (22% vs. 16%). Although multifocal PSMs were numerically more common in the hinotori group than in the da Vinci group (26% vs. 14%), the overall distribution of PSM locations did not differ significantly between the groups (overall *p* = 0.762).

**TABLE 3:** Perioperative and pathological outcomes of da Vinci and hinotori‐assisted radical prostatectomy in the propensity score‐matched cohort.

	da Vinci (*n* = 130)	hinotori (*n* = 65)	*p*
Operative time (min)	252 (217–284)	311 (256–353)	< 0.001
Console time (min)	188 (156–216)	232 (193–275)	< 0.001
Port placement time (min)	21 (17–26)	25 (22–30)	< 0.001
Docking time (min)	7 (5–10)	11 (9–15)	< 0.001
Time to console start (min)	29 (25–36)	37 (32–45)	< 0.001
Estimated blood loss (mL)	100 (50–215)	200 (100–450)	< 0.001
Blood transfusion, *n* (%)	0 (0)	0 (0)	N/A
Intraoperative complications, *n* (%)	2 (1)	0 (0)	0.553
Major postoperative complications, *n* (%)^a^	1 (1)	1 (1)	1.000
Postoperative hospital stay (days)	9 (8–10)	9 (8–10)	0.842
Pathological Gleason grade group, *n* (%)			0.647
Grade group 1	7 (5)	2 (3)	
Grade group 2	63 (48)	26 (40)	
Grade group 3	35 (27)	19 (29)	
Grade group 4	8 (6)	7 (11)	
Grade group 5	16 (12)	10 (15)	
Not available	1 (1)	1 (1)	
Pathological T stage, *n* (%)			0.240
pT0	0 (0)	1 (1)	
pT2	91 (76)	43 (66)	
pT3a	29 (16)	12 (19)	
pT3b	9 (7)	9 (13)	
pT4	1 (1)	0 (0)	
Positive surgical margin, *n* (%)			
Total	37 (28)	23 (35)	0.323
pT2 disease	17/91 (19)	13/43 (30)	0.182
pT3 or pT4 disease	20/39 (51)	10/21 (48)	1.000
Lymph node involvement, *n* (%)	5/66 (8)	4/34 (12)	0.485
Follow‐up after surgery (months)	68 (49–85)	25 (13–31)	< 0.001

*Note:* Values are presented as median (interquartile range [IQR]) or number (%).

^a^ Corresponding to Clavien–Dindo 3 or 4 complications within 30 days after surgery, as adjudicated by the attending surgeons.

**TABLE 4:** Distribution of positive surgical margin locations in the propensity score‐matched cohort.

Locations, *n* (%)	da Vinci (*n* = 37)	hinotori (*n* = 23)	*p*
Apex	12 (32)	6 (26)	
Bladder neck	7 (19)	3 (13)	
Anterior	6 (16)	2 (9)	
Posterolateral	6 (16)	5 (22)	
Posterior	1 (3)	1 (4)	
Seminal vesicle	0 (0)	0 (0)	
Multifocal^a^	5 (14)	6 (26)	
Overall distribution^b^			0.762

*Note:* Values are presented as number (%).

^a^ Defined as positive surgical margins at ≥ 2 locations.

^b^ Overall *p* value for the distribution of positive surgical margin locations between groups.

### 3.3 Predictors of Positive Surgical Margin

To further explore factors associated with PSM, univariable and multivariable logistic regression analyses were performed in the propensity score‐matched cohort (Table 5). In the univariable analysis, smaller prostate volume (odds ratio [OR] 0.970, 95% confidence interval [CI] 0.947–0.995, *p* = 0.017), higher clinical T stage (≥ cT2 vs. cT1; OR 2.160, 95% CI 1.110–4.210, *p* = 0.023), and later surgery year (OR 1.100, 95% CI 1.000–1.220, *p* = 0.048) were significantly associated with an increased risk of PSM. Multivariable analysis revealed that smaller prostate volume (OR 0.963, 95% CI 0.938–0.989, *p* = 0.005) remained an independent predictor of PSM, whereas neither the robotic platform (hinotori vs. da Vinci: OR 0.706, 95% CI 0.250–1.990; *p* = 0.512) nor surgery year (OR 1.170, 95% CI 0.990–1.370; *p* = 0.060) was independently associated with PSM.

**TABLE 5:** Univariable and multivariable analyses for predictors of positive surgical margin in the propensity score‐matched cohort.

Variables	Univariable			Multivariable		
	OR	95% CI	*p*	OR	95% CI	*p*
Age (years)	1.060	0.995–1.120	0.072	1.050	0.990–1.120	0.131
BMI (kg/m^2^)	0.998	0.893–1.120	0.974			
PSA (ng/mL)	1.050	0.996–1.100	0.089	1.040	0.980–1.110	0.229
Prostate volume, mL	0.970	0.947–0.995	0.017	0.963	0.938–0.989	0.005
Biopsy Gleason grade group (≥ 4 vs. ≤ 3)	1.210	0.580–2.510	0.616			
Clinical T stage (≥ cT2 vs. cT1)	2.160	1.110–4.210	0.023	1.410	0.680–2.940	0.363
Nerve‐sparing (Yes vs. No)	0.482	0.229–1.020	0.055	0.595	0.250–1.420	0.241
Robotic platform (hinotori vs. da Vinci)	1.410	0.746–2.670	0.290	0.706	0.250–1.990	0.512
Surgery year (per year)	1.100	1.000–1.220	0.048	1.170	0.990–1.370	0.060

Abbreviations: BMI, body mass index; CI, confidence interval; OR, odds ratio; PSA, prostate‐specific antigen.

### 3.4 Functional and Oncological Outcomes

**FIGURE 2:**
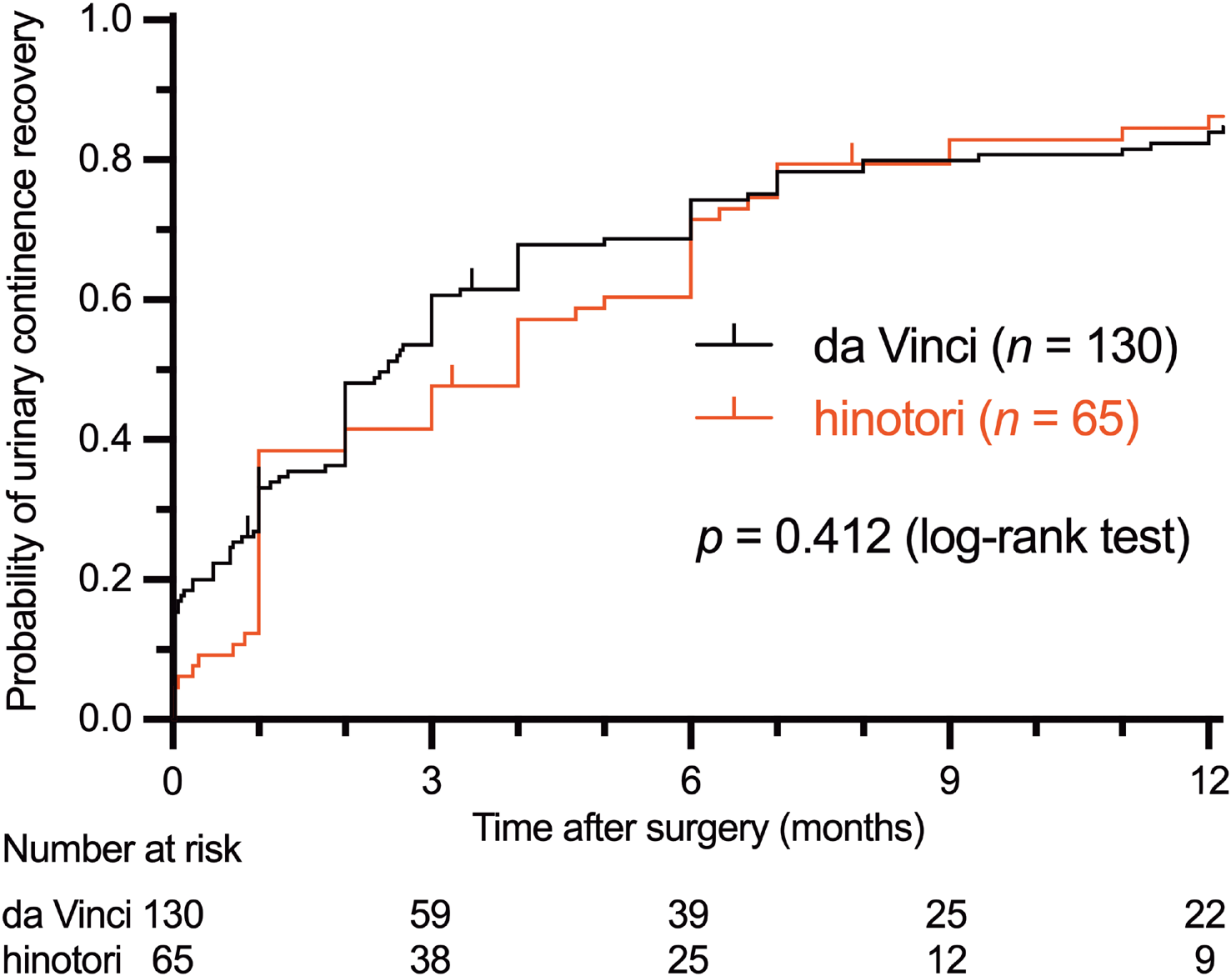
Comparison of time to recovery of urinary continence. Kaplan–Meier curves show the time to recovery of urinary continence after surgery in a propensity score‐matched cohort. The black line represents patients who underwent da Vinci‐assisted radical prostatectomy (*n* = 130), and the red line represents those who underwent hinotori‐assisted radical prostatectomy (*n* = 65). Urinary continence recovery was defined as the use of 0 or 1 safety pad per day. Differences between the groups were assessed using the log‐rank test.

**FIGURE 3:**
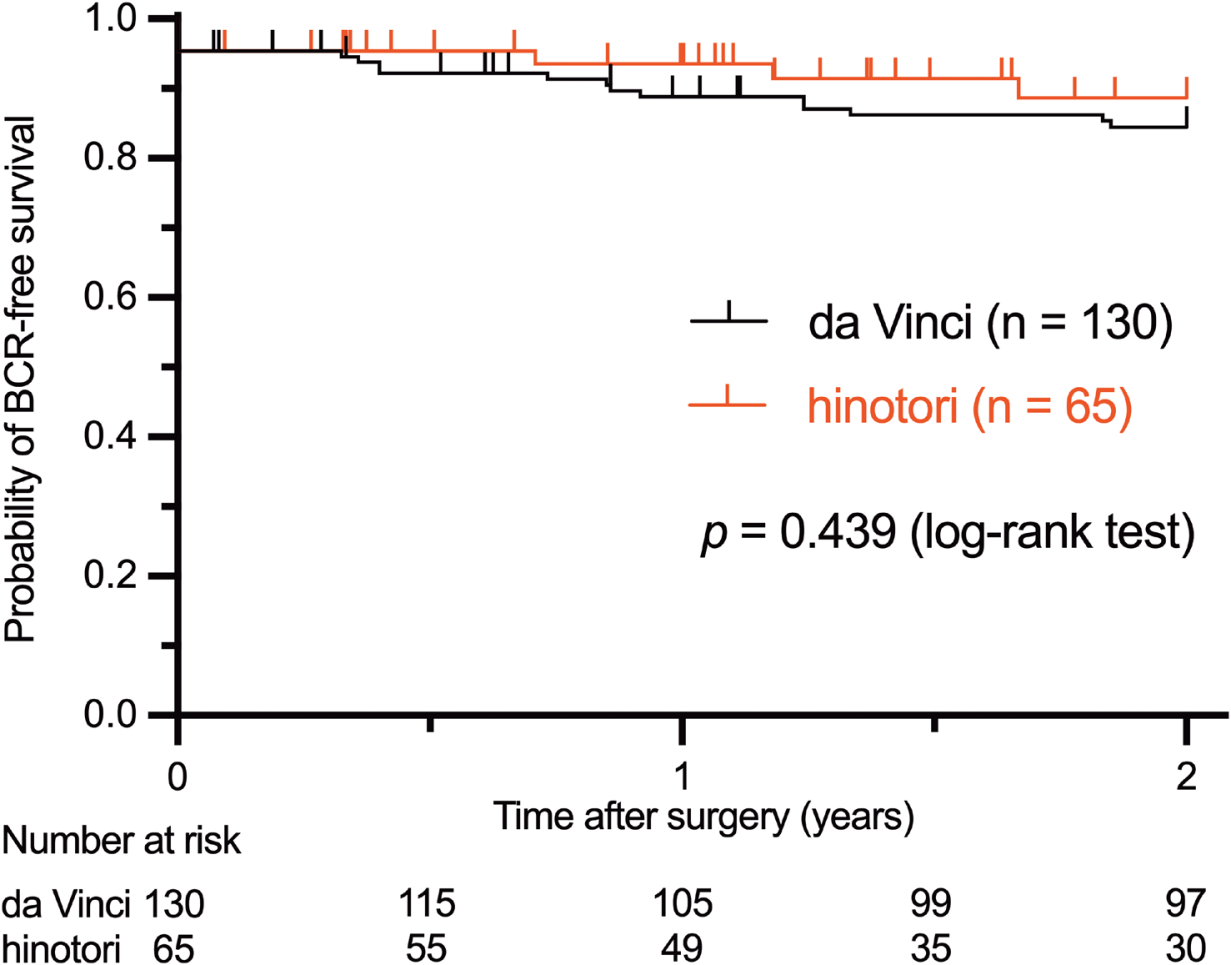
Comparison of biochemical recurrence (BCR)‐free survival with time‐aligned follow‐up. Kaplan–Meier curves show BCR‐free survival after surgery in the propensity score‐matched cohort with administrative censoring at 2 years (24 months). The black line represents patients who underwent da Vinci‐assisted radical prostatectomy (*n* = 130), and the red line represents those who underwent hinotori‐assisted radical prostatectomy (*n* = 65). BCR was defined as a postoperative serum prostate‐specific antigen level of ≥ 0.2 ng/mL confirmed by a second consecutive measurement. Differences between the groups were assessed using the log‐rank test.

The time to recovery of urinary continence did not differ significantly between the two groups (log‐rank *p* = 0.412; Figure 2). The cumulative continence recovery rates in the hinotori and da Vinci groups were 48% and 61% at 3 months, 72% and 74% at 6 months, and 87% and 85% at 12 months after surgery, respectively. BCR‐free survival after surgery is shown in Figure 3. To account for differences in follow‐up duration between groups, a time‐aligned analysis with administrative censoring at 2 years was performed. The BCR‐free survival curves appeared similar between the groups, with no significant difference observed (log‐rank *p* = 0.439). In landmark analysis, BCR‐free survival rates in the hinotori and da Vinci groups were 94% and 89% at 1 year and 89% and 85% at 2 years after surgery, respectively. The Kaplan–Meier curves for BCR‐free survival using the full follow‐up period demonstrated similar results (Figure S1).

## 4 Discussion

This propensity score‐matched retrospective study compared perioperative, pathological, functional, and early oncological outcomes between the hinotori and da Vinci robotic platforms in patients undergoing RARP in a real‐world cohort involving 17 surgeons with varying levels of RARP experience. The hinotori group showed longer operative and console times, longer setup‐related times, and greater EBL; however, no blood transfusions were required in either group, and perioperative safety profiles were comparable between platforms. No significant differences were observed in overall or stage‐specific PSM rates, anatomical PSM distribution, urinary continence recovery, or early BCR‐free survival between the hinotori and da Vinci groups. Multivariable analysis, adjusted for surgery year to account for potential era effects, further showed that robotic platform was not independently associated with PSM. These findings suggest that, although hinotori‐assisted RARP was associated with reduced perioperative efficiency during early adoption, surgical margin status, functional recovery, and early oncological outcomes were not clearly compromised compared with da Vinci‐assisted RARP.

In the present study, operative and console times were longer, and EBL was greater in the hinotori group than in the da Vinci group. Longer operative and console times with the hinotori system have also been reported in previous propensity score‐matched studies comparing hinotori‐ and da Vinci‐assisted RARP [7, 8], as well as in a recent systematic review [12]. In addition to these previously recognized trends, the present study further demonstrated that setup‐related times, including port placement time, docking time, and time to console start, were also significantly prolonged in the hinotori group. These findings suggest that the increased operative time was not solely attributable to console performance but may also reflect workflow adaptation during platform implementation. Several factors may explain these findings. First, all hinotori‐assisted procedures in this study were performed during the early institutional adoption period of the hinotori system. Second, platform‐specific operability may have contributed. Hinotori has unique structural features, such as eight‐axis robotic arms and software‐based trocar positioning; however, differences in console handling and surgical workflow compared with the da Vinci system have been reported [13]. In particular, the absence of a hand clutch function has been reported to be associated with prolonged operative time [14], whereas system updates, including the introduction of a hand clutch, have been linked to improved efficiency [15], suggesting that earlier versions of the system may have required additional adaptation. Third, surgeon‐related factors should be considered. In the propensity score‐matched cohort, RARP procedures were performed by 17 surgeons overall, including 12 surgeons who performed hinotori‐assisted procedures, and only 25 of 65 hinotori cases were performed by expert surgeons. Given that a minimum of 40 cases has been suggested to achieve basic proficiency in RARP [16], the distribution of hinotori cases across surgeons with varying levels of experience may have influenced operative performance. Taken together, the longer operative and setup‐related times and greater EBL observed in the hinotori group likely reflected a combination of institutional adoption, workflow adaptation, surgeon experience, and platform‐specific operability rather than a single factor. Importantly, despite these differences, blood transfusion was not required in either group, and complication rates and length of hospital stay were comparable between groups, suggesting that acceptable perioperative safety can be maintained during early clinical implementation when standardized surgical techniques are applied.

The PSM rate tended to be higher in the hinotori group than in the da Vinci group; however, no statistically significant differences were observed in overall or stage‐specific PSM rates between the two platforms (35% vs. 28%, *p* = 0.323; 30% vs. 19%, *p* = 0.182; and 48% vs. 51% for overall, pT2, and pT3 or pT4 disease, respectively). Although the PSM rate in the present study was slightly higher than that reported in a previous systematic review (overall 15% [range 6.5%–32%], pT2 9% [4%–23%], and pT3 37% [29%–50%]) [17], PSM is known to be influenced by tumor‐related factors, and differences in case mix may partly account for this result. In our multivariable analysis, smaller prostate volume was identified as an independent predictor of PSM, whereas the use of the hinotori system itself was not associated with an increased risk of PSM. These findings are consistent with previous RARP studies, in which multiple factors—including prostate size, PSA level, pathological T stage, pathological Gleason score, and surgeon experience—have been reported to influence margin status [18, 19]. A notable feature of the present study is the evaluation of anatomical PSM distribution in addition to overall PSM rates. The prostatic apex has been reported as the most common site of PSM, followed by the posterolateral region, likely reflecting the anatomical complexity of the apex and the technical challenges of dissection near the neurovascular bundles [19]. To our knowledge, few studies have examined PSM location in platform‐based comparisons; therefore, this is the first study to compare anatomical PSM distribution between hinotori‐ and da Vinci‐assisted RARP. In both groups, the apical and posterolateral margins were the most frequent sites of PSM, with no significant difference in the overall distribution. These findings suggest that hinotori‐assisted RARP did not show an apparent platform‐specific pattern of margin positivity in key anatomical regions.

Functional outcomes were similar between the hinotori and da Vinci systems, as postoperative recovery of urinary continence did not differ between the two platforms. The 12‐month continence recovery rates were 87% in the hinotori group and 85% in the da Vinci group. This result is consistent with findings from a recent systematic review showing that 12‐month urinary continence rates after RARP range from approximately 86.7%–95.1%, depending on the definition of continence (pad‐free or ≤ 1 pad per day) [20]. These findings suggest that the introduction of the hinotori system did not appear to adversely affect functional recovery after RARP. Regarding early oncological outcomes, BCR‐free survival was evaluated using a time‐aligned analysis with administrative censoring at 2 years to account for differences in follow‐up duration between groups. The BCR‐free survival curves appeared similar between the hinotori and da Vinci groups (log‐rank *p* = 0.439). In landmark analysis, the cumulative BCR‐free survival rates in the hinotori and da Vinci groups were 94% and 89% at 1 year and 89% and 85% at 2 years after surgery, respectively. However, because the follow‐up period in the hinotori group remained relatively short, longer follow‐up is required before drawing conclusions regarding long‐term oncological outcomes between the two platforms.

Several limitations of this study should be acknowledged. First, its retrospective design, single‐center setting, and relatively small sample size may have limited the generalizability of the findings. Second, although propensity score matching was used to reduce selection bias, residual confounding could not be completely eliminated. In particular, surgeon‐related factors, including surgeon allocation, experience, and case volume, were not incorporated into the propensity score model. Although surgeon distribution and expert status were described, surgeon‐specific effects and individual learning curves could not be fully adjusted for. Third, detailed preoperative tumor location data, such as MRI findings and biopsy core distribution, were not incorporated into the analysis, which may have influenced PSM risk. Fourth, the follow‐up period was shorter in the hinotori group. Although a time‐aligned analysis with administrative censoring at 2 years and landmark analyses were performed, longer follow‐up is required to evaluate long‐term oncological outcomes. Fifth, functional outcomes were limited to urinary continence, and sexual function outcomes, such as erectile function, were not evaluated. Finally, all hinotori‐assisted procedures were performed during the early institutional adoption period of the hinotori system. Therefore, the longer operative and setup‐related times observed in the hinotori group may reflect institutional workflow adaptation and surgeon‐team familiarization rather than the intrinsic performance of the platform itself.

In conclusion, hinotori‐assisted RARP was associated with longer operative, console, and setup‐related times and greater EBL than da Vinci‐assisted RARP. However, perioperative safety, pathological outcomes including PSM rates and anatomical distribution, urinary continence recovery, and early BCR‐free survival were not clearly compromised in this propensity score‐matched real‐world cohort.

## Author Contributions

All authors contributed to the conception and design of the study. Data curation, methodology, formal analysis, and resource acquisition were performed by M.Ni. and A.W. Additional data collection and investigation were performed by T.Y., S.K., M.Na., K.K., K.Y., and K.J. M.Ni. wrote the first draft of the manuscript. The manuscript was reviewed and edited by A.W. and T.Y. under the supervision of S.K. All authors have commented on the previous versions of the manuscript and have read and approved the final manuscript. All authors agree with the content of the manuscript.

## Funding

The authors have nothing to report.

## Ethics Statement

The study was approved by the Institutional Review Board of Shiga University of Medical Science Hospital (No. R2017‐034). Informed consent was obtained in the form of opt‐out on the website of Shiga University of Medical Science Hospital.

## Conflicts of Interest

The authors declare no conflicts of interest.

## Supporting information


**Figure S1:** Comparison of biochemical recurrence (BCR)‐free survival using full follow‐up data. Kaplan–Meier curves show BCR‐free survival after surgery in a propensity score‐matched cohort using the full available follow‐up period. The black line represents patients who underwent da Vinci‐assisted radical prostatectomy (*n* = 130), and the red line represents those who underwent hinotori‐assisted radical prostatectomy (*n* = 65). BCR was defined as a postoperative serum prostate‐specific antigen level of ≥ 0.2 ng/mL confirmed by a second consecutive measurement. Differences between the groups were assessed using the log‐rank test.

## Data Availability

The data that support the findings of this study are available from the corresponding author upon reasonable request.
