# Supplementary figures and images for: Comparison of Surgical Outcomes Between the Hinotori and da Vinci Platforms in Robot‐Assisted Radical Prostatectomy: A Propensity Score‐Matched Study
Source: Asian J Endosc Surg. 2026 Jun 15;19(1):e70326. doi: 10.1111/ases.70326 (PMC13266425; doi:10.1111/ases.70326)

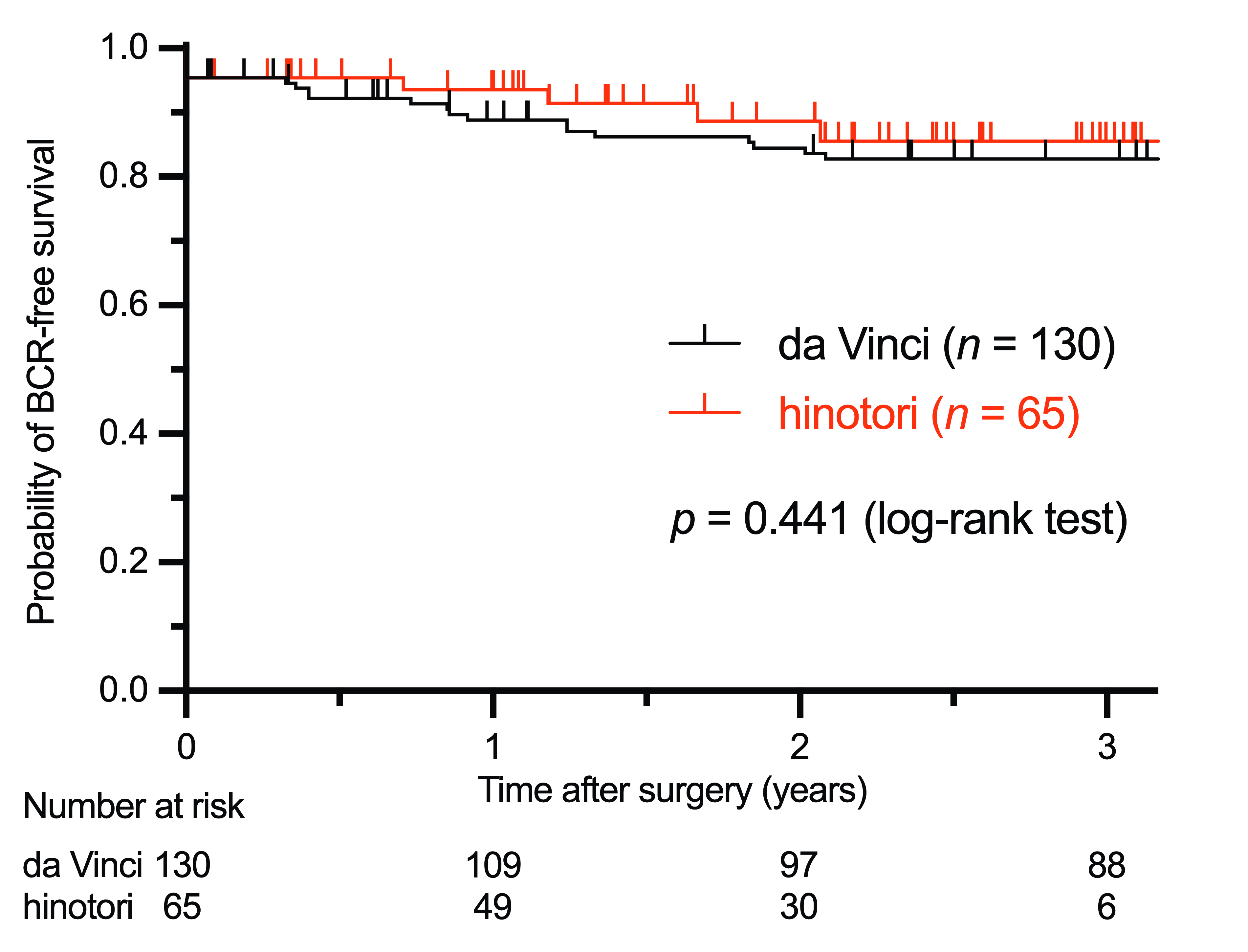

Supplement: Supplementary file 1 — Figure S1: Comparison of biochemical recurrence (BCR)‐free survival using full follow‐up data. Kaplan–Meier curves show BCR‐free survival after surgery in a propensity score‐matched cohort using the full available follow‐up period. The black line represents patients who underwent da Vinci‐assisted radical prostatectomy (n = 130), and the red line represents those who underwent hinotori‐assisted radical prostatectomy (n = 65). BCR was defined as a postoperative serum prostate‐specific antigen level of ≥ 0.2 ng/mL confirmed by a second consecutive measurement. Differences between the groups were assessed using the log‐rank test. [file ASES-19-e70326-s001.tiff]
